# Detection of SARS-CoV-2 in young children attending day-care centres in Belgium, May 2020 to February 2022

**DOI:** 10.2807/1560-7917.ES.2022.27.21.2200380

**Published:** 2022-05-26

**Authors:** Liesbet Van Heirstraeten, Esra Ekinci, Mathias Smet, Matilda Berkell, Laura Willen, Jasmine Coppens, An Spiessens, Basil Britto Xavier, Christine Lammens, Jan Verhaegen, Pierre Van Damme, Herman Goossens, Philippe Beutels, Veerle Matheeussen, Stefanie Desmet, Heidi Theeten, Surbhi Malhotra-Kumar

**Affiliations:** 1 Laboratory of Medical Microbiology, Vaccine and Infectious Disease Institute, University of Antwerp, Wilrijk, Belgium; 2 Centre for the Evaluation of Vaccination, Vaccine and Infectious Disease Institute, University of Antwerp, Wilrijk, Belgium; 3 Laboratory of Clinical Microbiology, Antwerp University Hospital, Edegem, Belgium; 4 Reference Centre for Pneumococci, University Hospitals Leuven, Leuven, Belgium; 5 Centre for Health Economics Research and Modelling Infectious Diseases, University of Antwerp, Wilrijk, Belgium

**Keywords:** SARS-CoV-2, carriage, Delta, Omicron

## Abstract

Presence of SARS-CoV-2 was monitored in nasopharyngeal samples from young children aged 6−30 months attending day-care centres (DCCs) in Belgium from May 2020−February 2022. SARS-CoV-2 carriage among DCC children was only detected from November 2021, after emergence of Delta and Omicron variants, in 9 of the 42 DCCs screened. In only one DCC, two children tested positive for SARS-CoV-2 at the same sampling time point, suggesting limited transmission of SARS-CoV-2 in Belgian DCCs among young children during the studied period.

Prevalence of severe acute respiratory syndrome coronavirus 2 (SARS-CoV-2) in young children (> 6 months) and their role in transmission remains unclear. Most SARS-CoV-2 infections in young children are asymptomatic or characterised by mild clinical symptoms [1], and consequently, young children are also less often tested than adolescents and adults [2,3]. At the onset of the coronavirus (COVID-19) pandemic in 2020, we introduced the collection of a nasopharyngeal (NP) swab for detection of SARS-CoV-2 in the child population attending day-care centres (DCCs) in Belgium. We investigated carriage of SARS-CoV-2 from May 2020 to February 2022 in young children attending 100 DCCs in Belgium, to gain insight on their role in SARS-CoV-2 transmission.

## Screening in day-care centres

The collection was undertaken as an additional sample in the framework of an ongoing study that started in 2016 to screen for pneumococcal serotype carriage in Belgian day-care children aged 6−30 months following sequential shifts in the pneumococcal conjugate vaccine programme [4]. Our initial screening from 2 to 12 March 2020 did not find any young children positive for SARS-CoV-2 in the DCC network [5]. This study included NP samples collected during 3 timeframes: May−June 2020 (95 samples), November 2020−May 2021 (751 samples), and 17 November 2021−8 February 2022 (299 samples) (Table 1). SARS-CoV-2 positivity among young children was detected only in the last timeframe from November 2021 up to February 2022 (Table 1).

**Table 1:** Overview of nasopharyngeal samples (n = 1,145) and day-care centres (n = 100) included per collection period, Belgium, May 2020–February 2022

Variables	Collection period		
	May–June 2020	November 2020–May 2021	November 2021–February 2022
Nasopharyngeal samples (n= 1,145)			
Total obtained	95	751	299
SARS-CoV-2-positive young children	0	0	11
Day-care centres (n= 100)			
Total included	12	95	42
Day-care centres with SARS-CoV-2-positive young children	0	0	9

SARS-CoV-2: severe acute respiratory syndrome coronavirus 2.

## Nasopharyngeal sample analysis

Two NP swab samples from each child, one from each nostril, were obtained with minitip flocked swabs (FLOQSwabs, COPAN); one swab was stored in DNA/RNA Shield medium (Zymo Research) and used for detection of SARS-CoV-2, while the other swab was stored in skim milk-glucose-glycerol (STGG) to enable screening for other respiratory pathogens, i.e. *Streptococcus pneumoniae* as part of the ongoing carriage study. Samples were transported on dry ice and stored at −80 °C until further analysis. 

RNA was extracted from 200µL of the DNA/RNA Shield sample using MagMAX Viral/Pathogen II Nucleic Acid Isolation Kit (ThermoFisher Scientific) on KingFisher Flex Purification System (ThermoFisher Scientific). Subsequently, reverse-transcriptase Real-Time PCR (RT-PCR) targeting SARS-CoV-2 was performed using QuantStudio 5 Real-Time PCR instrument (ThermoFisher Scientific) and TaqPath COVID-19 CE-IVD RT-PCR kit (ThermoFisher Scientific), targeting three regions (S protein, N protein, and ORF1ab) of the SARS-CoV-2 virus. Data analysis was performed using FastFinder Analysis software (UgenTec, Hasselt, Belgium). 

Whole genome sequencing (WGS) of the SARS-CoV-2 genome was conducted by preparation of multiplexed libraries with the Illumina COVIDSeq kit (Illumina Inc.) using a Zephyr G3 NGS Robot followed by 2 x 74 bp paired-end sequencing on a NextSeq 500/550 instrument (Illumina Inc.). Raw data quality assessment was performed using FastQC, followed by quality trimming with TrimGalore v. 0.6.7, reference mapping against the SARS-CoV-2 genome (GenBank: NC_045512.2) using the CLC Genomics Workbench v.9.5.3 (Qiagen). Consensus sequences were extracted, and clade and lineage assignment performed using Nextclade (https://clades.nextstrain.org) and Pangolin (https://pangolin.cog-uk.io), respectively.

## SARS-CoV-2 detection in young children

Carriage of SARS-CoV-2 among DCC children was only detected during the third timeframe of the study; SARS-CoV-2 was not detected in the 849 samples collected during the two preceding timeframes (May−June 2020 and November 2020−May 2021). A total of 299 NP samples were collected from young children attending 42 different DCCs (26 DCCs in Flanders, 12 in Wallonia and 4 in Brussels) during the third timeframe from November 2021 till February 2022. In 11 (3.7%) samples, the SARS-CoV-2 virus was detected by RT-PCR (Table 2). Six of the SARS-CoV-2 positive samples were collected during November−December 2021, where four showed presence of all three tested genes (ORF1ab, S protein, and N protein) and were confirmed by WGS to belong to the SARS-CoV-2 21J/Delta variant of concern (VOC) (Phylogenetic Assignment of Named Global Outbreak (Pango) lineage designation B.1.617.2; sublineages AY.43.3, AY.121, AY.4 and AY.43). The other five positive samples were collected during January−February 2022, and all were characterised by a negative RT-PCR for the S gene target, a so called ‘S gene drop-out’. Four of these were confirmed to belong to the 21K/Omicron VOC clade (Pango lineage designation B.1.1.529, subvariants BA.1 and BA.1.1) by WGS (Table 2). For the remaining three samples, the coverage was insufficient to determine the variant by WGS, and Cq values for the ORF1ab, N and S protein gene targets were high or undetected (Cq > 32, Table 2).

**Table 2:** Characteristics of SARS-CoV-2-positive young children in day-care centres, Belgium, November 2021–February 2022 (n = 11)

Child	Collection period	Age (months)	Number of children sampled in same DCC (n)	Region	Rhinitis^a^	Number of household members (n)	Parent/household member with symptoms^b^	RT-PCR (Cq values)			WGS		
								ORF1ab	N protein	S protein	Pango lineage	Pangolin version	Nextstrain clade (variant)
1	Nov–Dec 2021	17	10	Flanders	Yes	2	No	29.3	29.4	28.1	AY.43.3	3.1.19	21J (Delta)
2	Nov–Dec 2021	9	8	Brussels	Yes	3	Yes	25.9	25.4	30.7	AY.121	3.1.19	21J (Delta)
3	Nov–Dec 2021	12	8	Brussels	Yes	3	No	14.4	14.2	12.9	AY.4	3.1.19	21J (Delta)
4	Nov–Dec 2021	9	8	Brussels	Yes	2	Yes	36.0	UD	32.6	NA	NA	NA
5	Nov–Dec 2021	26	7	Flanders	Yes	4	Yes	32.5	32.2	UD	NA	NA	NA
6	Jan–Feb 2022	14	4	Flanders	Yes	2	Yes	26.8	26.1	UD	BA.1	3.1.20	21K (Omicron)
7	Nov–Dec 2021	17	9 + 3^c^	Flanders	No	3	No	18.8	17.8	17.5	AY.43	3.1.19	21J (Delta)
8	Jan–Feb 2022	19	9 + 3^c^	Flanders	Yes	4	No	20.1	19.2	UD	BA.1	3.1.20	21K (Omicron)
9	Jan–Feb 2022	19	7	Flanders	No	3	No	19.7	19.1	UD	BA.1.1	3.1.20	21K (Omicron)
10	Jan–Feb 2022	23	4	Wallonia	Yes	3	No	34.1	32.4	UD	NA	NA	NA
11	Jan–Feb 2022	11	6	Wallonia	Yes	4	No	28.6	28.7	UD	BA.1	3.1.20	21K (Omicron)

DCC: day-care centre; NA: not applicable; pango: Phylogenetic Assignment of Named Global Outbreak; SARS-CoV-2: severe acute respiratory syndrome coronavirus 2; WGS: whole genome sequencing: UD: undetermined.

^a^ Rhinitis in children was defined as coughing and/or a runny nose and was registered at the time of sampling.

^b^ Parent/household member reported rhinitis, sore throat, cough, fever, muscle ache, headache or decreased appetite within 2 weeks of the sample collection.

^c^ Two different sampling dates: nine children sampled Nov–Dec 2021; three other children sampled Jan–Feb 2022.

The age of cases ranged between 9 and 26 months (median: 17); four were female, seven were male and two of the parents were healthcare workers, one with and one without symptoms. Three children who tested positive for SARS-CoV-2 attended DCCs in Brussels, six children were in Flanders and the remaining two were in Wallonia. Two children that were SARS-CoV-2-positive at the same sampling time point in November attended the same DCC in Brussels; one child was found to carry a Delta variant, while for the other child, the viral variant could not be determined because of low sequencing coverage. In Flanders, two children who attended the same DCC both tested positive for SARS-CoV-2, but at different time points, i.e. in November and February, and for different viral variants, Delta and Omicron. 

SARS-CoV-2 positivity was thus detected in children attending nine of the 42 screened DCCs between November 2021 and February 2022, and in only one DCC, two of the eight screened children tested positive for SARS-CoV-2 at the same sampling time point. For all included DCCs where SARS-CoV-2 was detected, three to 10 children had been screened simultaneously in each DCC at the same time point. Our results suggest limited transmission between young children in day-care centres in Belgium. 

Of the 11 SARS-CoV-2-positive children, nine had mild symptoms (rhinitis) at the time of sampling. However, of the 288 SARS-CoV-2-negative children, 136 (47.2%) also had rhinitis, indicating that rhinitis was not a discriminatory symptom. The 11 SARS-CoV-2-positive children were living together with at least two household members; for four children, one of the household members showed symptoms such as a runny nose, sore throat, cough, fever, muscle aches, headache, or loss of appetite within 2 weeks before the sample collection and one of them was a healthcare worker (Table 2). None of the parents travelled abroad within 2 weeks before the sampling.

## Discussion

We detected SARS-CoV-2 positivity in children attending DCCs only after the emergence of Delta and Omicron variants, and in a context of moderate to very high prevalence rates (16–46%; November 2021–February 2022) in the general population in Belgium [6]. These findings are in line with the increase in overall infectiousness and transmissibility, including in young children, as reported after the emergence of these VOCs [3]. The circulating VOCs and the level of vaccine uptake in older age groups were found to be very important for the relative prevalence of SARS-CoV-2 in young children [3,7]. A recent study in the United States (US) showed that young children (especially children between ages 0−4 years) also have an important role in household transmission of the Omicron variant [8]. However, the role of children in transmission of SARS-CoV-2 in a non-household setting such as in a DCC is still unclear. In the US and South Africa, an increase in paediatric emergency visits and COVID-19 hospitalisations for young children was observed, which paralleled the increased circulation of the Omicron variant [9-11]. Overall, COVID-19 symptoms remain mild in children.

A potential driver of SARS-CoV-2 transmission is the viral load, which can be measured by quantification of the viral RNA levels using RT-PCR and determining the Cq values [12]. Other studies identified similar levels of SARS-CoV-2 RNA in samples from asymptomatic, pre-symptomatic and symptomatic individuals, as well as in adults and children [13-16]. Nevertheless, a faster viral clearance was found in asymptomatic individuals [17]. Cq values in our study ranged between 14 and 36, with no clear distinction between patients showing rhinitis or not.

Of note, only preventive measures regarding good hand hygiene and ventilation were implemented in included DCCs and there were no restrictions on contact between children and face masks for staff in contact with children were also not mandatory. Our findings suggest that transmission between children in day-care did not occur very frequently although children were tested only once; even upon detection of SARS-CoV-2, re-sampling and testing was not performed. Another limitation of the study was that not all children attending the DCCs nor the staff were tested, only children for whom parents provided informed consent were included in the study.

## Conclusion

Continued monitoring is needed to further elucidate the role of young children in the ongoing COVID-19 pandemic, especially in environments such as DCCs where close contact between young children could facilitate the spread of SARS-CoV-2. Such surveillance is necessary, particularly to guide public health interventions such as screening, vaccination, and temporary closure of DCCs.
